# Health-related Quality of Life and Problem Behavior After GH Cessation in Adults Born Small for Gestational Age: A 12-Year Follow-up Study

**DOI:** 10.1210/clinem/dgae425

**Published:** 2024-06-18

**Authors:** Demi Justine Dorrepaal, Manouk van der Steen, Maria de Ridder, Wesley Jim Goedegebuure, Anita Charlotte Suzanne Hokken-Koelega

**Affiliations:** Department of Pediatrics, Subdivision of Endocrinology, Erasmus University Medical Center-Sophia Children's Hospital, P.O. 2060, 3000 CB Rotterdam, The Netherlands; Department of Pediatrics, Subdivision of Endocrinology, Radboud University Medical Center, 6525 GA Nijmegen, The Netherlands; Department of Medical Informatics, Erasmus University Medical Center, 3015 CN Rotterdam, The Netherlands; Department of Pediatrics, Subdivision of Endocrinology, Erasmus University Medical Center-Sophia Children's Hospital, P.O. 2060, 3000 CB Rotterdam, The Netherlands; Department of Pediatrics, Subdivision of Endocrinology, Erasmus University Medical Center-Sophia Children's Hospital, P.O. 2060, 3000 CB Rotterdam, The Netherlands

**Keywords:** small for gestational age, growth hormone treatment, health-related quality of life, problem behavior, long-term follow-up

## Abstract

**Context:**

Long-term data regarding health-related quality of life (HRQoL) and problem behavior in adults born small for gestational age (SGA) who were treated with GH during childhood are lacking.

**Objective:**

To investigate longitudinal changes in HRQoL and problem behavior in adults born SGA during 12 years after cessation of childhood GH treatment (SGA-GH) and compare these with 3 control groups at age around 30 years.

**Participants:**

One hundred seventy-six SGA-GH adults and 3 untreated age-matched control groups: 50 born SGA with short stature (SGA-S), 77 born SGA with spontaneous catch-up growth to normal height (SGA-CU), and 99 born appropriate-for-gestational-age with normal height (AGA).

**Main Outcome Measures:**

HRQoL and problem behavior were assessed using the TNO-AZL Adults Quality of Life questionnaire and Adolescent Behavior Check List at 6 months and 2, 5, and 12 years after GH cessation. Data at 12 years after GH cessation were compared with 3 control groups.

**Results:**

During 12 years after GH cessation, HRQoL remained similar on 9 subscales in SGA-GH adults but decreased on 3 subscales (gross motor functioning, pain, sleep). Externalizing problem behavior decreased significantly, and internalizing problem behavior tended to decrease. SGA-GH and SGA-S adults had similar HRQoL and problem behavior. SGA-GH adults had, compared to AGA adults, similar HRQoL on 7 subscales, lower HRQoL on 5 subscales, and more internalizing and externalizing problem behavior. All SGA adults had lower HRQoL and more internalizing problem behavior than AGA adults. Adult height associated negatively with externalizing problem behavior, but the influence was small.

**Conclusion:**

During 12 years after GH cessation, HRQoL remained mostly similar and problem behavior decreased in SGA-GH adults. SGA-GH and SGA-S adults had similar HRQoL and problem behavior. All SGA adults had lower HRQoL and more internalizing problem behavior than AGA adults.

Short stature after being born small for gestational age (SGA) has been associated with a reduction in health-related quality of life (HRQoL) and more problem behavior in children and adults (1-5). HRQoL implies the appraisal of one's health status by the person themself (6, 7). HRQoL is multidimensional, including physical, emotional, and social aspects of well-being and functioning (8, 9). In children born SGA with persistent short stature, GH treatment increases adult height (10-12). During GH treatment, HRQoL and problem behavior were investigated.

HRQoL was not different in GH-treated children born SGA compared to untreated short children born SGA on the generic Child Health Questionnaire and on 3 of the 5 subscales of the disorder-specific TNO-AZL Children's Quality of Life questionnaire for short stature (13). Compared to a healthy reference population, HRQoL was similar in GH-treated children on 10 of 11 subscales of the Child Health Questionnaire (13). During GH treatment, internalizing problem behavior remained similar and comparable to Dutch peers, but externalizing problem behavior improved significantly to scores comparable with Dutch peers, while the improvement was related to change in height SD score (SDS) (14). Long-term data regarding HRQoL and problem behavior in adults born SGA who were treated with GH during childhood are lacking.

The primary objective of our study was to investigate longitudinal changes in HRQoL and problem behavior in adults born SGA from cessation of GH treatment at attainment of adult height until 12 years after cessation at around age 30 years (SGA-GH) and descriptively compare these longitudinal changes with age-related values of a reference group. We hypothesized that longitudinal HRQoL and problem behavior of SGA-GH adults would not change during the 12-years follow-up and would be similar compared to the reference group. The other primary objective was to compare HRQoL and problem behavior in SGA-GH adults at age around 30 years with age-matched untreated adults born SGA with persistent short stature (SGA-S) and adults born appropriate for gestational age (AGA). Based on previous studies that showed similar HRQoL and problem behavior in GH-treated children born SGA compared to untreated short children born SGA and healthy peers, we hypothesized that the HRQoL and problem behavior of SGA-GH adults would also be similar compared to SGA-S and AGA adults. The secondary objective was to compare HRQoL and problem behavior in all adults born SGA, including SGA-GH, SGA-S, and adults born SGA with spontaneous catch-up growth during childhood (SGA-CU), with AGA adults. In addition, we performed regression analyses to determine if adult height was associated with HRQoL and problem behavior at around age 30 years.

## Methods

### Study Design and Participants

The study population comprised 402 adults, of whom 176 (SGA-GH) had participated in Dutch GH trials during their childhood because of persistent short stature (height SDS < −2) after being born SGA (birthweight or birth length SDS < −2) (15). They were treated with GH until adult height (AH) attainment. Details about in- and exclusion criteria of the Dutch GH trials were previously reported (16). At AH, all SGA-GH adults were invited to participate in a follow-up study to assess HRQoL at 6 months, 2 years, 5 years, and 12 years and problem behavior at 2, 5, and 12 years after GH cessation. At the end of the 12-year follow-up period at around age 30 years, SGA-GH adults were compared with 3 GH-untreated control groups of similar age from the PROgramming factors for Growth And Metabolism study (17): (1) adults born SGA (birthweight or birth length SDS < −2) with persistent short stature (AH SDS < −2) (SGA-S), (2) adults born SGA with spontaneous catch-up growth to a normal AH (SDS > −1) (SGA-CU) and (3) adults born AGA (birthweight or birth length SDS > −1) with normal AH (SDS > −1) (AGA). SGA-S and SGA-CU adults were recruited after reviewing hospital records from several Dutch hospitals. Additionally, healthy AGA adults were randomly selected from different postsecondary educational levels. SGA-S adults had not participated in the Dutch GH trials as children, because pediatricians in the eastern part of The Netherlands were not involved in the Dutch GH trials. Therefore, these individuals made the most appropriate control group of GH-untreated adults with persistent short stature. The Medical Ethics Committee of Erasmus University Medical Center (Rotterdam, The Netherlands) approved the study, and all participants gave written informed consent.

### Assessment of Clinical Characteristics

Clinical characteristics at birth were obtained via birth records of hospitals. At a patient age of around age 30 years, we measured standing height to the nearest 0·1 cm (Harpenden stadiometer; Holtain, Crymmyth, UK) and weight to the nearest 0·1 kg on a digital scale (Servo Balance KA-20-150S; Servo Berkel Prior, Katwijk, The Netherlands). We expressed AH and adult weight as SDS, adjusted for sex, based on references for Dutch adults, using Growth Analyser Research Calculation Tools (https://growthanalyser.org/) (18). Body composition was measured by a dual-energy x-ray absorptiometry scan. All measurements were made with the same machine (Lunar Prodigy; GE Healthcare, Chalfont St Giles, UK) and software (enCORE software version 14.1), with daily quality assurance. The intra-assay coefficient of variation was 0·41% to 0·88% for fat mass (19). Fat mass index was calculated as fat mass in kilograms divided by length in meters squared (kg/m^2^). Adults provided information regarding socioeconomic status, presence of a chronic medical condition or psyciatrisch/mental condition, and lifestyle factors at around age 30 years through a structured questionnaire. Yearly income (low: <€10.000; middle: €10.000-50.000; high: >€50.000) and highest completed level of education (low: lower secondary education; middle: upper secondary education or postsecondary nontertiary education; high: bachelor's degree or higher) were used to determine socioeconomic status. Information about the presence (yes vs no) of a chronic physical condition or psychiatric/mental condition was collected and interpreted by one investigator (D.D.). Examples of a psychiatric/mental condition were anxiety disorder, attention deficit hyperactivity disorder, and depression. Examples of a chronic physical condition were asthma, migraine, thyroid disorder, and previous cancer diagnose.

### HRQoL

Subjects completed the generic and validated TNO-AZL Adults Quality of Life (TAAQoL) to assess HRQoL (20). The TAAQoL consists of 45 items and contains 12 subscales to determine someone's health status, arbitrarily divided into physical aspects like gross motor functioning, fine motor functioning, pain, daily activities, sex, and vitality and emotional/social aspects like sleep, social contacts, happiness, depressive mood, anger, daily activities, sex, vitality, and cognition. Questions about daily activities, sex, and vitality have both physical and social/emotional aspects of HRQoL; for that reason we added them to both the physical and social/emotional aspect of HRQoL. Most items consist of 2 questions: the first question assesses whether a participant has experienced a health status limitation in the last couple of weeks. If present, the second question scores the emotional feeling about this limitation. The scale scores are obtained by combining both responses into 1 HRQoL score per item, adding item scores within scales and transforming crude scale scores to a 0 to 100 scale. Higher scores indicate a better HRQoL. This questionnaire specifically offers the subjects the possibility to differentiate between their functioning and the way they feel about it. Based on Dutch population data, Cronbach alpha, a reliability coefficient to measure the internal consistency of the TAAQoL scale scores, ranged from 0.72 to 0.90, indicating that comparisons on the group level are justified (20). The psychometric properties, reliability, and validity of this questionnaire were also satisfactory (21). Longitudinal HRQOL of our study group was descriptively compared with the values of the TAAQoL reference group in 2 subsequent age ranges, namely 16 to 25 (n = 351) and 26 to 35 years (n = 912) (see Supplemental Material).

### Problem Behavior

Subjects completed the Adolescent Behavior Check List (ABCL) to assess problem behavior (22). The questionnaire consists of 113 questions on specific problem behavior, scored on a 3-point Likert scale (0 indicating absent behavior; 2 indicating behavior is frequently present). This questionnaire is one of the most widely used dimensional rating scales of psychopathology, as it rates behavior on 3 main scales (total, externalizing, and internalizing problem behavior) and 8 subscales (withdrawn behavior, somatic complaints, anxious/depressed behavior, social problems, thought problems, attention problems, delinquent behavior, and aggressive behavior). Externalizing problem behavior reflects conflicts with other people and with social mores. Internalizing problem behavior mainly reflects problems within the self (anxiety, self-perception, depression, somatic complaints without known medical cause) and withdrawal from social contacts. Raw scores are continuous and were transformed into standardized T-scores for males and females separately, based on a reference population that had a mean of 50 and a SD of 10. Higher scores indicate more problem behavior. This questionnaire has been studied extensively in clinical and community populations (23). The longitudinal problem behavior of our study group was descriptively compared with the ABCL reference group in the age range 18 to 35 years (n = 585) (see Supplemental Material).

### Contentment With Adult Height

Subjects were also asked by questionnaire about their own subjective contentment (yes/no) with their adult height at around age 30 years. Of the 331 subjects, 95% (n = 314) completed this questionnaire.

### Statistical Analyses

Clinical characteristics are presented as mean and SD or percentages. ANOVA, unpaired *t*-test, and Chi-square test were used to compare the clinical characteristics among the 4 groups. During the follow-up from GH cessation to 12 years thereafter, 71 of 176 SGA-GH adults dropped out because of the time-consuming aspect of the study, resulting in 105 participants at around age 30 years. Longitudinal assessment of HRQoL and problem behavior in SGA-GH adults was therefore performed using repeated measurements analyses. HRQoL, subscales sleep, pain, vitality, and happiness were log transformed and analyzed using linear mixed model (LMM) with an unstructured covariance matrix. Subscales anger and cognition were analyzed using Poisson LMM with a random intercept for the anger subscale and a random intercept and slope for the cognition subscale (24). Subscales gross motor functioning, social contacts, daily activities, and sex were analyzed using the Hurdle–Poisson model with a random intercept (25). Subscale fine motor functioning could not be analyzed using repeated measurements analysis because most SGA-GH adults reported the maximum score of 100 and this score did not change over time. Only descriptive results were available for this subscale. Longitudinal HRQoL was descriptively compared with the TAAQoL reference group in 2 subsequent age ranges. Problem behavior was analyzed using LMM with an unstructured covariance matrix for externalizing and internalizing problem behavior. Longitudinal problem behavior was descriptively compared with the ABCL reference group. The Mann–Whitney U test was used to compare HRQoL and problem behavior at age 30 years between SGA-GH and SGA-S adults and between SGA-GH and AGA adults (primary objectives). When a significant difference among the 4 groups was found on a subscale by the Kruskal–Wallis test, the Mann–Whitney U test was performed to investigate which SGA group(s) significantly differed from the AGA adults and in addition if all adults born SGA (SGA-GH, SGA-S, and SGA-CU adults) differed from the AGA adults (secondary objectives). Multiple linear regression analyses were performed in the total study population at around age 30 years, first to investigate if adult height was associated with HRQoL and problem behavior. In addition, we investigated if birth characteristics (birth weight SDS and birth length SDS) were associated with HRQoL and problem behavior in the total study population. The interaction term birth length SDS * adult height SDS was added to the models because the study groups had been selected on birth length SDS and adult height SDS, in order to ensure that the effect of these variables were modeled correctly. Subsequently, we added other potential determinants in the regression models: sex, age, fat mass index, chronic physical condition (yes vs no), mental/psychiatric condition (yes vs no), educational level (low, middle, high), hours of physical exercise (never, ≤ 2 hours/week, ≥ 3 hours/week), smoking (never, current smoker, former smoker), alcohol consumption (never, ≤ 3 units/week, ≥ 4 units/week) and illicit drug use (yes vs no). The potential determinants were only added to the models if they had a significant association with HRQoL or problem behavior in the univariate analyses; only significant potential determinants remained in the final models. There was no overlap between chronic physical condition and mental/psychiatric condition in the total study population. Statistical analyses were performed using SPSS version 28.0 for Windows and R version 4.2.1 for Windows. Results were regarded statistically significant when *P*-value <.05.

## Results

### Clinical Characteristics

Table 1 shows the clinical characteristics of the total study population at birth, the 176 SGA-GH subjects at the start and cessation of GH treatment, and the 331 subjects at around age 30 years. In the SGA-GH subjects, mean (SD) age at start of GH treatment was 7.0 (2.5) years, mean GH-treatment duration 8.5 years, (2.4) and mean age at GH cessation 15.9 years (1.4). Mean age at follow-up visit around age 30 years was 28.6 years (3.4) in SGA-GH, 31.8 years (3.3) in SGA-S, 32.7 years (2.5) in SGA-CU, and 32.7 years (2.6) in AGA adults (*P* < .001). Birth length and birth weight were different between the SGA groups and the AGA group, as this was part of the inclusion criteria. Mean AH SDS in SGA-GH was −1.6 SDS, which was higher than in SGA-S (−2.4 SDS, *P* < .001) but lower than in SGA-CU (−0.2 SDS, *P* < .001) and AGA (0.4 SDS, *P* < .001). There was a significant difference in educational level (*P* < .001), income (*P* < .001), and fat mass index (*P* = .04) between the groups. No differences between the groups were found for chronic medical condition, mental/psychiatric condition, and lifestyle factors (smoking, alcohol consumption, illicit drug use, and hours of exercise). The clinical characteristics at birth were similar for the 71 SGA-GH subjects lost to follow-up compared to the 105 SGA-GH subjects around age 30 years, but the latter started at a later age with GH treatment (7.4 vs 6.4 years, *P* = .02), stopped earlier (15.6 vs 16.4 years, *P* < .001), and had a shorter GH-treatment duration (7.7 vs 9.5 years, *P* < .001) compared to the subjects lost to follow-up.

**Table 1.** Clinical characteristics

	Study group	Comparison groups			*P*-value
	SGA-GH	SGA-S	SGA-CU	AGA	
	mean (SD)	mean (SD)	mean (SD)	mean (SD)	
n subjects	176	50	77	99	
At birth					
Gestational age, weeks	36.6 (3.9)*^a,c^*	37.8 (3.0)	36.6 (3.2)	38.7 (2.7)	**<.001**
Birth length SDS	−3.4 (1.5)*^b,c^*	−3.0 (1.3)	−2.5 (1.1)	0.1 (1.0)	**<.001**
Birth weight SDS	−2.5 (1.1)*^c^*	−2.2 (0.9)	−2.3 (1.0)	0.3 (1.0)	**<.001**
At start GH treatment					
n (%) females	89 (50.6)	NA	NA	NA	
Age, years	7.0 (2.5)	NA	NA	NA	
At GH cessation					
Age, years	15.9 (1.4)	NA	NA	NA	
GH duration, years	8.5 (2.4)	NA	NA	NA	
At 12 years after GH cessation or at around age 30 years					
n subjects	105	50	77	99	
n (%) females	59 (56.2)	31 (62.0)	43 (55.8)	55 (55.6)	.883
Age, years	28.6 (3.4)*^a,b,c^*	31.8 (3.3)	32.7 (2.5)	32.7 (2.6)	**<.001**
Adult height SDS	−1.6 (1.0)*^a,b,c^*	−2.4 (0.6)	−0.2 (0.7)	0.4 (0.9)	**<.001**
Demographics					
Fat mass index (kg/m2)	7.12 (3.1)	8.32 (3.8)	7.90 (3.2)	6.75 (3.2)	**.04**
Chronic physical condition (%)	19.0	20.8	18.9	14.9	.81
Psychiatric condition (%)	14.0	12.5	12.5	6.4	.36
Education (%)	*^b,c^*				**<.001**
Low	21.2	10.4	12.2	4.3	
Medium	44.5	50.0	32.4	22.6	
High	34.3	39.6	55.4	73.1	
Income (%)	*^b,c^*				**<.001**
Low	14.1	10.6	7.5	3.4	
Medium	82.4	80.9	68.7	67.4	
High	3.5	8.5	23.9	29.2	
Smoking (%)					.40
Never	63.4	72.9	62.7	73.1	
<10 cigarettes/day)	12.8	10.4	17.3	8.6	
≥10 cigarettes/day)	11.9	10.4	10.7	4.3	
History	11.9	6.3	9.3	14.0	
Alcohol use (%)					.19
None	16.7	16.7	9.3	12.8	
<1 unit/week	30.4	27.0	38.7	27.7	
1–3 units/week	31.4	39.6	17.3	30.9	
4–6 units/week	14.7	12.5	25.3	17.0	
≥1 unit/day	6.8	4.2	9.3	11.7	
Illicit drug use (%)					.21
Any drug	14.9	10.4	21.3	10.8	
Cannabis	10.5	8.0	10.4	3.0	
Ecstasy	3.8	0	10.4	7.1	
Cocaine	4.8	0	6.5	4.0	
Exercise (%)					.67
Never	36.0	33.3	26.7	20.4	
<1 hour/week	4.0	2.1	2.7	5.4	
1–2 hours/week	29.0	35.4	33.3	38.7	
3–5 hours/week	21.0	20.8	29.3	23.7	
>5 hours/week	10.0	8.3	8.0	11.8	

Continuous variables are presented as mean (SD); socioeconomic scales and lifestyle factors are presented as percentages. Bold *P*-values are considered significant differences between groups.

Abbreviations: AGA, appropriate for gestational age with normal height; SDS, SD score; SGA, small for gestational age; SGA-CU, small for gestational age with spontaneous catch-up growth to normal height; SGA-S, small for gestational age with short stature.

^*a*^ *P* < .05 between SGA-GH and SGA-S.

^*b*^ *P* < .05 between SGA-GH and SGA-CU.

^*c*^ *P* < .05 between SGA-GH and AGA.

### Longitudinal HRQoL in SGA-GH Adults

**Figure 1.**
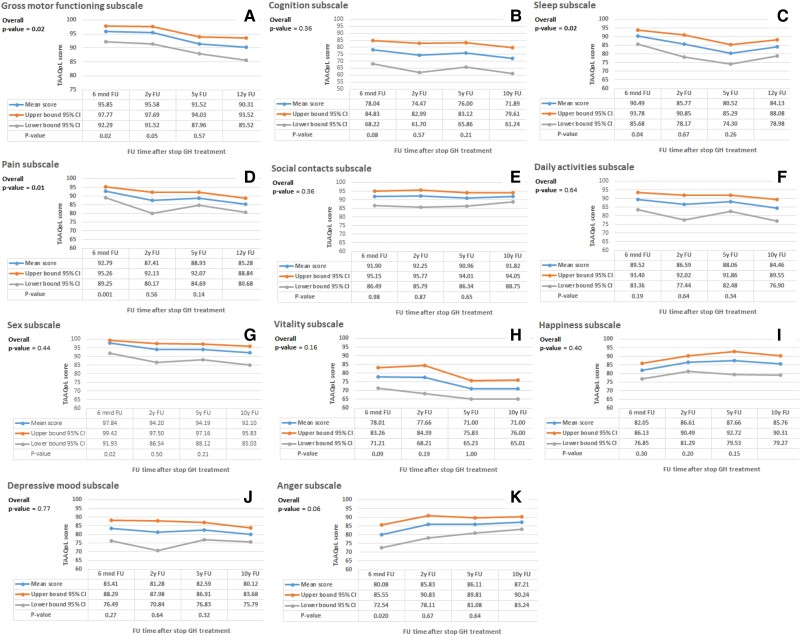
Longitudinal assessment of HRQoL in SGA-GH adults. The figure presents 11 of the 12 subscales of the TAAQoL questionnaire to assess HRQoL; it was completed by subjects at 6 months, 2 years, 5 years, and 12 years after GH cessation. The reference follow-up moment was 12 years after GH cessation. The blue line shows the mean score at the different follow-up moments. The orange line shows the upper limit of the 95% CI and the grey line shows the lower limit of the 95% CI of the mean score. Bold *P*-values indicate significant change over time. Abbreviations: CI, confidence interval; HRQoL, health-related quality of life; SGA, small for gestational age; TAAQoL, TNO-AZL Adults Quality of Life.

Figure 1 shows the estimated means (95% confidence interval) of the longitudinal HRQoL. Subscale fine motor functioning could not be analyzed because most (93%) SGA-GH adults scored the maximum score of 100, which did not change over time, indicating no longitudinal changes on this subscale. During 12 years after GH cessation, no significant changes in HRQoL were found on additionally 8 of the 12 subscales, namely, cognition, social contacts, daily activities, sex, vitality, happiness, and depressive mood and anger. HRQoL decreased on 3 subscales: gross motor functioning (*P* = .02) and pain (*P* = .01) (physical subscales) and sleep (*P* = .02) (social/emotional subscale). The TAAQoL reference group showed no major changes in HRQoL on these 3 subscales in either men and women (see Supplemental Material).

### Longitudinal Problem Behavior in SGA-GH Adults

**Figure 2.**
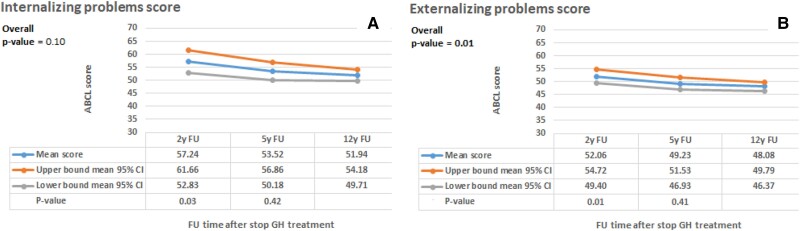
Longitudinal assessment of problem behavior in SGA-GH adults. The figure presents the internalizing and externalizing problem behavior scores of the ABCL questionnaire to assess problem behavior; it was completed by subjects at 2 years, 5 years, and 12 years after GH cessation. The reference follow-up moment was 12 years after GH cessation. The blue line shows the mean score at the different follow-up moments. The orange line shows the upper limit of the 95% CI, and the grey line shows the lower limit of the 95% CI of the mean score. Bold *P*-values indicate significant change over time. Abbreviations: ABCL, Adolescent Behavior Check List; CI, confidence interval; SGA, small for gestational age.

Figure 2 shows the estimated means (95% confidence interval) of the longitudinal problem behavior. Externalizing problem behavior decreased (*P* = .01) and internalizing problem behavior tended to decrease (*P* = .10) during the 12 years after GH cessation. Comparing these findings with those of the ABCL reference group showed that internalizing problem behavior remained higher than the reference group while externalizing problem behavior decreased to lower scores than the reference group in both men and women (see Supplemental Material).

### HRQoL of SGA-GH Adults Compared to Untreated Control Groups at Around Age 30 Years

Table 2 shows the HRQoL in SGA-GH adults at 12 years after GH cessation compared to the age-matched control groups. SGA-GH and SGA-S adults had a similar HRQoL based on all 12 subscales. SGA-GH adults had a similar HRQoL as AGA adults on 7 subscales but a lower HRQoL on 5 subscales: pain (*P* = .03) and vitality (*P* = .01) (physical subscales) and cognition (*P* = .04), vitality (*P* = .01), happiness (*P* = .04), and anger (*P* = .007) (social/emotional subscales).

**Table 2.** Health-related quality of life at 12 years after GH cessation or at around age 30 years

	Study group	Comparison groups				
	SGA-GH	SGA-S	SGA-CU	AGA		All adults born SGA
	mean (SD)	mean (SD)	mean (SD)	mean (SD)	*P*-value	mean (SD)
Gross motor functioning*	91.2 (17.4)	94.9 (11.2)	90.8 (16.2)	95.5 (10.4)	.10	91.8 (16.0)
Fine motor functioning*	98.5 (4.8)	97.9 (6.7)*^b^*	97.2 (7.6)*^c^*	99.6 (1.8)	**.04**	97.9 (6.3)*^d^*
Cognition^	78.7 (23.9)*^a^*	83.7 (17.8)	77.1 (23.0)	84.1 (21.6)	.05	79.1 (22.6)
Sleep^	72.6 (24.6)	74.0 (19.3)	70.6 (24.1)	74.8 (25.2)	.20	72.1 (23.4)
Pain*	75.8 (22.4)*^a^*	78.1 (20.9)	72.9 (23.3)*^c^*	82.4 (15.8)	**.03**	75.2 (22.4)*^d^*
Social contacts^	91.9 (13.6)	96.1 (8.1)	91.3 (14.0)	92.8 (12.6)	.55	92.5 (13.0)
Daily activities*^	87.4 (20.4)	89.5 (18.8)	86.4 (18.7)	88.9 (16.5)	.72	87.4 (19.4)
Sex*^	91.3 (18.8)	92.4 (16.1)	83.9 (24.5)	91.4 (15.7)	**.04**	88.8 (20.8)
Vitality*^	64.5 (22.4)*^a^*	63.8 (20.5)*^b^*	63.5 (21.5)*^c^*	69.8 (19.1)	**.01**	64.0 (21.6)*^d^*
Happiness^	72.6 (19.9)*^a^*	74.6 (15.2)*^b^*	71.4 (21.1)*^c^*	77.8 (17.6)	**.03**	72.5 (19.5)*^d^*
Depressive mood^	81.6 (17.6)	80.5 (15.4)*^b^*	77.0 (20.2)*^c^*	83.3 (15.2)	**.04**	79.7 (18.3)*^d^*
Anger^	88.6 (15.8)*^a^*	91.2 (12.7)*^b^*	86.9 (17.8)*^c^*	93.4 (10.1)	**.03**	88.5 (16.0)*^d^*

The TNO-AZL Adults Quality of Life questionnaire was completed by subjects 12 years after GH cessation. Scores are presented as mean (SD). Higher scores indicate better health-related quality of life. Bold *P*-values are considered significant differences between the 4 groups.

Abbreviations: AGA, appropriate for gestational age with normal height; SGA, small for gestational age; SGA-CU, small for gestational age with spontaneous catch-up growth to normal height; SGA-S, small for gestational age with short stature.

^*^Subscale scoring physical health-related quality of life.

^^^Subscale scoring emotional/social health-related quality of life.

^*a*^ *P* < .05 between SGA-GH adults and AGA adults.

^*b*^ *P* < .05 between SGA-S adults and AGA adults.

^*c*^ *P* < .05 between SGA-CU adults and AGA adults.

^*d*^ *P* < .05 between all SGA adults and AGA adults.

The 4 groups differed significantly regarding fine motor functioning, pain, sex, and vitality (physical subscales) and regarding sex, vitality, happiness, depressive mood and anger (social/emotional subscales). Compared to AGA adults, SGA-S adults had a lower HRQoL on 5 subscales, like the SGA-GH adults, and SGA-CU adults had a lower HRQoL on 6 subscales. Taken together, all adults born SGA had a lower HRQoL than AGA adults regarding fine motor functioning (*P* = .01), pain (*P* = .004), and vitality (*P* = .001) (physical subscales) and vitality (*P* = .001), happiness (*P* = .004), depressive mood (*P* = .01), and anger (*P* = .002) (social/emotional subscales).

### Problem Behavior of SGA-GH Adults Compared to Untreated Control Groups at Around Age 30 Years

Table 3 shows problem behavior of SGA-GH adults at 12 years after GH cessation compared to the age-matched control groups. SGA-GH and SGA-S adults had similar internalizing and externalizing problem behavior. SGA-GH adults had more internalizing (*P* = .004) and externalizing (*P* = .02) problem behavior than AGA adults. Also, SGA-S and SGA-CU adults had more internalizing problem behavior compared to AGA adults. Taken together, all adults born SGA had more internalizing (*P* < .001) problem behavior compared to AGA adults.

**Table 3.** Problem behavior at 12 years after GH cessation or at around age 30 years

	Study group	Comparison groups				
	SGA-GH	SGA-S	SGA-CU	AGA		All adults born SGA
	mean (SD)	mean (SD)	mean (SD)	mean (SD)	*P*-value	mean (SD)
Depressed/anxious	55.1 (7.2)^*a*^	56.4 (8.9)^*b*^	54.4 (6.6)	52.8 (5.4)	**.02**	55.1 (7.4)^*d*^
Withdrawn	55.1 (6.1)	54.9 (7.1)	54.8 (6.4)	54.1 (5.4)	.52	55.0 (6.4)
Somatic complaints	55.6 (7.7)^*a*^	55.8 (8.3)^*b*^	56.0 (7.0)^*c*^	52.8 (4.1)	**.01**	55.8 (7.5)^*d*^
Thought problems	54.6 (6.3)^*a*^	54.2 (6.9)	53.0 (4.5)	52.8 (4.5)	.15	53.9 (5.9)
Attention problems	55.5 (5.5)^*a*^	54.6 (5.8)	54.4 (4.5)	53.6 (4.5)	.08	54.9 (5.3)
Aggressive behavior	52.8 (3.8)	52.8 (3.7)	52.7 (4.1)	52.2 (3.6)	.28	52.8 (3.8)
Rule-breaking behavior	53.0 (3.7)^*a*^	51.8 (2.1)	52.3 (3.2)	51.7 (2.6)	.20	52.5 (3.3)
Intrusive	52.1 (3.6)	52.0 (3.3)	51.6 (3.2)	51.4 (3.3)	.22	51.9 (3.4)
Total scores						
Internalizing problems	51.9 (10.7)^*a*^	53.2 (11.5)^*b*^	51.2 (11.1)^*c*^	47.5 (9.6)	**.01**	51.9 (11.0)^*d*^
Externalizing problems	48.0 (8.3)^*a*^	48.1 (6.8)	47.2 (8.2)	44.9 (8.5)	.07	47.7 (7.9)
Total problems	48.7 (9.9)^*a*^	49.5 (8.8)^*b*^	48.1 (8.2)	45.3 (8.8)	**.047**	48.6 (9.1)^*d*^

The Adolescent Behavior Check List questionnaire was completed by subjects 12 years after GH cessation. Scores are presented as mean (SD). Lower scores indicate less problem behavior. Bold *P*-values are considered significant differences between groups.

Abbreviations: AGA, appropriate for gestational age with normal height; SGA, small for gestational age; SGA-CU, small for gestational age with spontaneous catch-up growth to normal height; SGA-S, small for gestational age with short stature.

^*a*^ *P* < .05 between SGA-GH adults and AGA adults.

^*b*^ *P* < .05 between SGA-S adults and AGA adults.

^*c*^ *P* < .05 between SGA-CU adults and AGA adults.

^*d*^ *P* < .05 between all adults born SGA and AGA adults.

### Contentment With Adult Height at Around Age 30 Years

Significantly more SGA-GH adults (86%) were content with their adult height compared to SGA-S adults (60%) (*P* < .001), but SGA-GH adults were less content compared to AGA adults, 86% vs 95%, respectively (*P* = .04). SGA-S adults (60%) were also less content with their adult height compared to AGA adults (95%) (*P* < .001), while most of the SGA-CU (91%) and AGA adults (95%) were content with their adult height (*P* = .31).

### Multiple Linear Regression for HRQoL in Adults Around Age 30 Years

Linear regression analyses were performed in the total study population at around age 30 years, first to investigate if adult height was associated with HRQoL. We chose the vitality subscale as the dependent variable because it includes both physical HRQoL and emotional/social HRQoL. In the univariate (unadjusted) regression, adult height SDS was positively associated with vitality (B = 3.1, *P* = .001, adjusted R^2^ = 0.03) (not shown in Table 4). In addition, we investigated if birth characteristics were associated with HRQoL. Birth weight SDS was positively associated with vitality (B = 1.6, *P* = .045, adjusted R^2^ = 0.01) (also not shown in Table 4), but birth length was not associated. Subsequently, we investigated if other potential determinants, as mentioned in the statistical analyses, were associated with HRQoL. The final model (Table 4), including only potential determinants that remained significant, shows that, adjusted for all variables included in the model, adult height SDS and birth characteristics were not associated with vitality. Of the potential determinants, alcohol consumption and ≥3 hours/week of physical exercise were positively associated with vitality while chronic physical condition, mental/psychiatric condition, and current and former smoking were negatively associated, together explaining 24.6% of the variation in vitality score at around age 30 years (*P* < .001). For example, the final model shows that current smoking was associated with a decreased vitality score of 15.1 (score scale 0-100).

**Table 4.** Multiple linear regression analyses for HRQoL vitality score, internalizing problem behavior, and externalizing problem behavior at 12 years after GH cessation or at around age 30 years

		HRQoL—vitality subscale			Externalizing problem behavior			Internalizing problem behavior		
		B (SE)	β	*P*-value	B (SE)	β	*P*-value	B (SE)	β	*P*-value
*P* intercept		64.4 (6.3)		<.001	37.0 (2.6)		<.001	39.9 (4.3)		<.001
Adult height SDS		2.7 (1.7)	.16	.11	−0.9 (0.4)	−.26	.02	−1.6 (1.1)	−.20	.13
Birth weight SDS		−1.9 (1.5)	−.14	.19				0.3 (0.8)	.04	.73
Birth length SDS		0.9 (1.3)	−.70	.49				−0.3 (0.8)	−.06	.65
BL SDS * AH SDS		0.1 (0.5)	.02	.88				−0.1 (0.3)	−.06	.68
Potential determinants										
Chronic physical condition		−10.0 (3.2)	−.17	**.002**						
Mental/psychiatric condition		−10.5 (3.6)	−0.15	**.007**	7.1 (1.6)	.28	**<.001**	8.6 (2.4)	.26	**<.001**
Fat mass index					0.3 (0.2)	.14	**.03**	0.47 (0.2)	.16	**.04**
Educational level	Low				Ref					
	Medium				−0.9 (1.6)	−.06	.56			
	High				−4.0 (1.5)	−.26	**.01**			
Smoking	Never	Ref								
	Current	−15.1 (3.1)	−.28	**<.001**						
	Former	−9.0 (3.9)	−.13	**.02**						
Alcohol consumption	None	Ref						Ref		
	≤3 units/week	14.3 (3.7)	.31	**<.001**				−4.5 (2.3)	−.21	**.05**
	≥4 units/week	24.4 (4.4)	.48	**<.001**				−5.3 (2.6)	−.23	**.04**
	Illicit drug use				4.8 (1.4)	.22	**<.001**			
Physical exercise	Never	Ref								
	≤2 hours/week	1.1 (3.1)	.03	.71						
	≥3 hours/week	6.9 (3.2)	.14	**.03**						
	Adjusted R^2^ (*P*-value)	0.246 (<0.001)			0.204 (<0.001)			0.143 (<0.001)		

HRQoL scores range from 0 to 100; higher scores indicate better HRQoL. Problem behavior scores are standardized T-scores, which have a mean of 50 and a SD of 10 in the reference population; higher scores indicate more problem behavior. Bold *P*-values indicate a significant association with HRQoL vitality score, internalizing problem behavior or externalizing problem behavior at 12 years after GH cessation or at around age 30 years.

Abbreviations: AH, adult height; B (SE), unstandardized coefficient and SE; BL, birth length; β, standardized coefficient; HRQoL, health-related quality of life; SDS, SD score.

### Multiple Linear Regression for Problem Behaviour in Adults Around Age 30 Years

Linear regression analyses were performed in the total study population at around age 30 years, first to investigate if adult height was associated with problem behavior. In the univariate (unadjusted) regression, adult height SDS was negatively associated with externalizing (B = −1.5, *P* < .001, adjusted R^2^ = 0.05) and internalizing problem behavior (B = −2.1, *P* < .001, adjusted R^2^ = 0.06) (not shown in Table 4). In addition, we investigated if birth characteristics were associated with problem behavior. Birth weight SDS (B = −0.8, *P* = .049, adjusted R^2^ = 0.01) and birth length SDS (B = −0.7, *P* = .04, adjusted R^2^ = 0.01) were negatively associated with internalizing problem behavior (not shown in Table 4) but not associated with externalizing problem behavior. Subsequently, we investigated if other potential determinants, as mentioned in the statistical analyses, were associated with problem behavior. The final model (Table 4) of externalizing problem behavior, including only potential determinants that remained significant, shows that, adjusted for all variables included in the model, adult height SDS was negatively associated and birth characteristics were not associated with externalizing problem behavior. Of the potential determinants, high educational level was negatively associated while mental/psychiatric condition, fat mass index, and illicit drug use were positively associated, together explaining 20.4% of the variation in externalizing problem behavior. The final model of internalizing problem behavior, including only potential determinants that remained significant, shows that, adjusted for all variables included in the model, adult height SDS and birth characteristics were not associated with internalizing problem behavior. Of the potential determinants, ≥ 4 units/week of alcohol was negatively associated while mental/psychiatric condition and fat mass were positively associated, together explaining 14.3% of the variation in internalizing problem behavior (*P* < .001).

## Discussion

This is the first study that longitudinally assessed HRQoL and problem behavior in SGA-GH adults during 12 years after GH cessation. In addition, HRQoL and problem behavior in SGA-GH adults at 12 years after GH cessation at around age 30 years were compared to 3 untreated age-matched control groups: SGA-S, SGA-CU, and AGA adults. HRQoL remained mostly similar during the 12 years after GH cessation, except for a decrease in gross motor functioning, pain, and sleep. In the reference group, no major changes in HRQoL were found on these 3 subscales. Longitudinal externalizing problem behavior significantly decreased to scores lower than the reference group, while internalizing problem behavior tended to decrease but remained higher than the reference group. At around age 30 years, SGA-GH and SGA-S adults had similar HRQoL and problem behavior. SGA-GH and AGA adults had mostly similar HRQoL, but SGA-GH adults had lower HRQoL regarding cognition, pain, vitality, happiness, and anger, and they had more internalizing and externalizing problem behavior than AGA adults. More internalizing problem behavior was also found in the other SGA groups. Adult height was not associated with HRQoL and internalizing problem behavior and was negatively associated with externalizing problem behavior, but the influence was small.

HRQoL is important as a patient-reported outcome because it reflects the subjective perception of health. HRQoL can be divided into physical and social/emotional HRQoL. Longitudinal physical HRQoL did not change regarding fine motor functioning, daily activities, sex, and vitality but decreased regarding gross motor functioning and pain, albeit weakly significant. These 2 subscales showed no major changes in the TAAQoL reference group (age range 16-35 years). The decrease in gross motor functioning in SGA-GH adults might be related to the fact that adults born SGA have a lower lean body mass compared to AGA adults at around age 30 years (17). The decrease in HRQoL subscale pain in SGA-GH adults, indicating more pain, is probably not related to cessation of GH treatment but might be explained by the fact that SGA-GH adults had a lower educational level and probably more often a physical profession compared to AGA adults, which could have increased back pain or pain in joints and muscles. One study reported longitudinal HRQoL in a group of untreated SGA adults with a short and normal stature from age 20 to 32 years compared to AGA controls and found also that physical HRQoL significantly decreased compared to the AGA controls (2).

Longitudinal social and emotional HRQoL did not change regarding cognition, social contacts, daily activities, sex, vitality, happiness, depressive mood, and anger but decreased regarding sleep, indicating more sleep problems, albeit weakly significant. The sleep subscale showed no major changes in the TAAQoL reference group. Sleep is largely influenced by life stage and social context, and the decrease in sleep seems unrelated to previous GH treatment and its cessation. The study reporting longitudinal HRQoL in a group of untreated SGA adults with a short and normal stature from age 20 to 32 years compared to AGA controls showed that mental HRQoL significantly increased compared to the AGA controls (2). However, comparing our results with those authors’ findings is difficult because they used different questionnaires with different subscales to assess HRQoL.

Externalizing problem behavior, reflecting conflicts with other people, significantly decreased during the 12 years of follow-up to scores lower than the reference group, while internalizing problem behavior, reflecting problems within the self, tended to decrease but remained higher than the reference group. A possible explanation for the decrease in externalizing problem behavior might be a more mature and structured daily life with more responsibilities (work, family, children) with increasing age, which could reduce problem behavior. We could not compare our findings with literature data as no studies investigated longitudinal problem behavior in SGA or AGA adults. Thus, problem behavior decreased during the 12 years after GH cessation, which was more positive than we had hypothesized.

Our other primary objective was to compare HRQOL and problem behavior in SGA-GH adults at 12 years after GH cessation with age-matched untreated SGA-S and AGA adults at around age 30 years. Previous studies showed similar HRQoL and problem behavior in GH-treated children born SGA compared to untreated short children born SGA and to healthy peers (13, 14). In line with these results, we found similar physical HRQoL, emotional/social HRQoL, and problem behavior in SGA-GH and SGA-S adults. A possible explanation could be that SGA-S adolescents and adults have learned to cope with their persistent short stature. In support of this explanation, it has been reported that coping variables like behavioral disengagement, venting emotions, and denial tend to be stronger predictors than illness perception variables, like illness identity, cause, and consequences, on outcomes of HRQoL, depression, and anxiety in physical health conditions (26). These findings show that coping may prevent a lower HRQoL and might explain our finding of similar HRQoL in SGA-GH and SGA-S adults.

SGA-GH and AGA adults had mostly similar HRQoL. In SGA-GH adults, however, physical HRQoL was lower regarding pain and vitality, and emotional/social HRQoL was lower regarding cognition, vitality, happiness, and anger, albeit weakly significant. These findings are in line with our multiple regression analyses showing that adult height and birth characteristics had no influence on HRQoL while other determinants, like a physical or mental/psychiatric condition, had a significant influence on HRQoL.

Regarding problem behavior, SGA-GH adults had similar externalizing and internalizing problem behavior compared to SGA-S adults but more problem behavior than AGA adults. We could not compare our results with literature data because no studies compared HRQoL or problem behavior in SGA-GH adults with SGA-S or AGA adults. The results of our regression analyses suggest that a higher prevalence of mental/psychiatric conditions and a lower educational level in SGA-GH adults compared to AGA adults could be contributing factors for more externalizing and internalizing problem behavior.

All adults born SGA, previously GH treated and nontreated, had in general lower HRQoL compared to AGA adults. They had a lower physical HRQoL regarding fine motor functioning, pain, and vitality and lower emotional/social HRQoL regarding vitality, happiness, depressive mood, and anger. All adults born SGA had more internalizing problem behavior compared to AGA adults, suggesting that a lower HRQoL and more internalizing problem behavior are probably more related to the underlying condition of being born SGA than to GH treatment. The study that reported longitudinal HRQoL from age 20 to 32 years in untreated SGA adults with a short and normal stature compared to AGA controls also compared HRQoL at age 32 years and found lower physical HRQoL and more problems with work or other daily activities in adults born SGA due to physical health problems (2). We found a higher percentage of chronic physical conditions, like asthma, migraine, and thyroid disorders, in all SGA groups compared to the AGA group. In addition, SGA adults had lower levels of education and lower income compared to AGA adults, which is in line with Strauss et al, who also found lower income in SGA compared to AGA adults (27).

A higher percentage of SGA-GH adults were content with their adult height compared to SGA-S adults, but they were less content compared to AGA adults. This can be explained by the fact that adult height in SGA-GH adults was higher compared to SGA-S adults but lower compared to AGA adults. Adequate counseling of expectations before the start of GH treatment is important.

Our regression analyses showed that adult height SDS was not associated with HRQoL at around age 30 years. Population studies showed conflicting results regarding the influence of adult height on HRQoL (28, 29). Christensen et al found that height was significantly associated with HRQoL, with the strongest association in the subgroup with a height SDS < −2.0 (R^2^ = 6.1%) (28). In contrast, Coste et al found that height was a very weak predictor of HRQoL in the general population with adjusted R^2^ statistics <0.2% (29). Our regression analyses showed that adult height SDS was not associated with internalizing problem behavior while it was associated with externalizing problem behavior at around age 30 years, but it had only a small influence. No other studies investigated the influence of adult height on problem behavior at around age 30 years.

Birth characteristics and GH treatment were not associated with HRQoL in our final regression models. The presence of a chronic physical condition, a mental/psychiatric condition, and current or (former) smoking were negatively associated with HRQoL, while alcohol consumption and ≥3 hours/week of physical exercise were positively associated. In the study of Christensen et al, female sex and the presence of a chronic physical condition were also negatively associated with HRQoL, in addition to older age, higher weight, and lower social class (28). The positive association between HRQoL and alcohol consumption might be explained by the fact that alcohol is mostly consumed during social events with friends, which has a positive influence on HRQoL. We found more externalizing problem behavior in the case of a mental/psychiatric condition, higher fat mass index, and illicit drug use and less in the case of a high educational level. More internalizing problem behavior was found in the case of a mental/psychiatric condition and higher fat mass index and less in the case of ≥4 units of alcohol a week. These findings cannot be compared with the literature as no other studies investigated determinants of problem behavior around age 30 years in adults born SGA or AGA adults.

It has been established that GH treatment normalizes adult height in most short children born SGA (10-12, 30, 31). GH treatment also increases lean body mass, while fat mass, lipid levels, and blood pressure decrease, which are positive health benefits (32-35). Our research group has studied metabolic and cardiovascular health and cerebrovascular safety both during and after cessation of GH treatment in subjects born SGA (15, 16, 36-38). At 12 years after GH cessation at around age 30 years, SGA-GH adults had similar metabolic, cardiovascular, and cerebrovascular health as untreated short adults born SGA and normal statured adults born AGA, with the exception of a lower lean body mass and higher serum lipid levels, but that was present in all adults born SGA (17). These findings show that it is beneficial and safe to treat short SGA children with GH, even up to 12 years after GH cessation.

This is the first prospective study that longitudinally assessed HRQoL and problem behavior in SGA-GH adults from GH cessation until 12 years thereafter and compared HRQoL and problem behavior in SGA-GH adults around age 30 years with appropriate untreated control groups. There are, however, limitations. For the longitudinal assessment of HRQoL and problem behavior in SGA-GH adults, we had no longitudinal information on our untreated control groups. Instead, we show the HRQoL and problem behavior data of the reference groups at similar ages as the SGA-GH adults. Due to the explorative nature of our study, it was unrealistic to adjust for multiple testing, but it means that weak associations or differences could be due to chance. As a result, these weak associations and differences need to be interpreted with caution and do not allow strong conclusions. Although our total study population was relatively large, the proportion of SGA-S adults was smaller due to the low prevalence of persistent short stature after SGA birth (±0.1% of all live-born children) (39). This was complicated further, because most children with short stature were treated with GH for AH improvement during the last 25 years. We, therefore, also compared our results of the SGA-GH adults with the larger group of age-matched AGA adults. For logistical reasons, the SGA-S subjects did not receive GH treatment because only children in the western part of The Netherlands were invited to participate in the Dutch GH trials. This makes bias due to unmeasured confounding highly unlikely.

In conclusion, during 12 years after GH cessation, HRQoL remained mostly similar in SGA-GH adults, while externalizing problem behavior decreased and internalizing problems tended to decrease. At around age 30 years, SGA-GH and SGA-S adults had similar HRQoL and problem behavior. SGA-GH and AGA adults had mostly similar HRQoL, but SGA-GH adults had lower HRQoL regarding cognition, pain, vitality, happiness, and anger, and they had more problem behavior. This was also true for the other untreated SGA groups, suggesting that lower HRQoL and more internalizing problem behavior are probably more related to the underlying condition of being born SGA than to GH treatment. Adult height was negatively associated with externalizing problem behavior, but the influence was small.

## Data Availability

Some or all datasets generated during and/or analyzed during the current study are not publicly available but are available from the corresponding author on reasonable request.
